# Differences between Roma and non-Roma in how social support from family and friends helps to overcome health care accessibility problems

**DOI:** 10.1186/s12939-015-0165-z

**Published:** 2015-04-14

**Authors:** Daniela Bobakova, Zuzana Dankulincova Veselska, Ingrid Babinska, Daniel Klein, Andrea Madarasova Geckova, Lydia Cislakova, the HEPA-META team

**Affiliations:** Graduate School Kosice Institute for Society and Health, Safarik University, Kosice, Slovak Republic; Department of Health Psychology, Medical Faculty, Safarik University, Trieda SNP 1, 040 11 Kosice, Slovak Republic; Olomouc University Social Health Institute, Palacky University in Olomouc, Univerzitni 22, Olomouc, 771 11 Czech Republic; Department of Epidemiology, Regional Public Health Authority with the seat in Kosice, Ipelska 1, 040 11 Kosice, Slovak Republic; Institute of Mathematics, Science Faculty, Safarik University, Jesenna 5, 040 01 Kosice, Slovak Republic; Institute of Epidemiology – Medical Faculty, Safarik University, Srobarova 2, 041 80 Kosice, Slovak Republic

**Keywords:** Roma, Ethnicity, Health care accessibility problems, Social support, Slovakia

## Abstract

**Background:**

Roma are the most deprived ethnic minority in Slovakia, suffering from discrimination, poverty and social exclusion. Problematic access to good quality health care as result of institutional and interpersonal discrimination affects their health; therefore, factors which affect health care accessibility of Roma are of high importance for public health and policy makers. The aim of this study was to explore the association between health care accessibility problems and ethnicity and how different levels of social support from family and friends affect this association.

**Methods:**

We used data from the cross-sectional HepaMeta study conducted in 2011 in Slovakia. The final sample comprised 452 Roma (mean age = 34.7; 35.2% men) and 403 (mean age = 33.5; 45.9% men) non-Roma respondents.

**Results:**

Roma in comparison with non-Roma have a more than 3-times higher chance of reporting health care accessibility problems. Social support from family and friends significantly decreases the likelihood of reporting health care accessibility problems in both Roma and non-Roma, while the family seems to be the more important factor.

**Conclusion:**

The worse access to health care of Roma living in so-called settlements seems to be partially mediated by social support. Interventions should focus on Roma health mediators and community workers who can identify influential individuals who are able to change a community’s fear and distrust and persuade and teach Roma to seek and appropriately use health care services.

## Introduction

Roma (so-called Gypsies) make up one of the largest minority populations in Europe. More than 400,000 Roma are estimated to be living in Slovakia, which represents 7.5% of Slovak population [1]. Fewer than half of all Roma (46.5%) live scattered among the majority population; the rest live either in urban concentrations within or on the outskirts of towns or villages (36.7%) or in separated or segregated Roma settlements (17%) [1]. The latter, in particular, are characterised by degrading living conditions. The poor housing of Roma often has no connection to a sewerage system and/or running water and is cut off from transport networks, etc. [1]. This “circle of failure” is closed by a low educational level that leads to low employment rates and results in a high dependency on welfare benefits passed from one generation to the next [2].

As mentioned above, Roma are the most deprived ethnic minority in Slovakia and suffer from discrimination, poverty and social exclusion [2-4]. These unfavourable conditions have a remarkable impact on the health of the Roma population compared with the majority population, as has been previously shown by several studies. Roma were found to have higher infant mortality rates, lower life expectancy and a higher frequency of health complaints [5,6]. Some studies have also shown poorer self-rated health in Roma compared with non-Roma [7-9]. Not only do Roma appear to have worse health, they also seem to face problems in accessing good quality health care [10]. Moreover, there might be an association between these two factors [11,12], which have not been adequately studied [10]. Roma seem to be systematically excluded from key aspects of health care, such as preventive, primary and specialised health services as well as pre- and postnatal health care [10] and instead increasingly rely on emergency services [10].

Roma appear to perceive their own health differently than the majority population [13,14]. The health of Roma is often perceived through the perspective of their families, which seems to be rather logical considering the tight social bonds within Roma families and communities [15]. Whether the family is doing fine or not might have an influence on an individual’s perception of his or her own health and well-being [15]. This is in line with the social capital perspective, which views networks of relationships as a valuable resource (social capital) for individuals. According to Putnam (2000) social capital consists of features such as networks, norms and trust that facilitate coordination and cooperation for mutual benefit and aid [16]. Social capital can be conceptualized at both the collective and individual level [17,18]. Collective social capital refers to characteristics of communities, workplaces or neighbourhoods, whereas individual social capital concerns elements related to social relationships of individuals [19]. Social network ties within family, friends and community provide social support and are thought to play a critical role in accessing and transferring resources, including material, information and knowledge [20]. Social support has been categorised in various ways, but in general it covers social interactions that “lead the subject to believe that he or she is cared for, loved, esteemed and a member of a network of mutual obligations” [21]. In line with this theoretical perspective, several studies have confirmed the role of social support in influencing health-care-seeking behaviour and treatment adherence [22-24]. Boateng et al. [23] in their study explored possible enablers and barriers in access to the Dutch health care system among Ghanaians in Amsterdam. Among the most frequently mentioned enablers they listed availability/accessibility of family support and community initiatives/cohesion as important factors. Community was mentioned to provide enlightenment on health issues, and family members provided support in direct contact with health professionals. In addition, Gotay and Wilson [24] in their summary of several studies focused on the association of social support with breast and cervical cancer screening in certain minority groups in the USA and found a connection between received social support and an increase in cancer screenings.

Problematic access to good quality health care as result of institutional and interpersonal discrimination of Roma affects their health [25]; therefore, factors which affect the health care accessibility of Roma are of high importance for public health and policy makers.

Thus, the aim of this study was to explore the mediating as well as moderating effect of social support on the association between health care accessibility problems and ethnicity.

## Methods

We used data from the cross-sectional HepaMeta study conducted in 2011 in Slovakia. This project aimed to map the prevalence of viral hepatitis B/C and metabolic syndrome in the population living in separated and segregated Roma settlements and to compare it with the occurrence of the same health indicators in the majority population in regard to the selected risk and protective factors of these health indicators.

### Sample and procedure

The highest concentrations of the Roma population in Slovakia can be found in the eastern part of the country [26]. Therefore, the target population was residents of Roma settlements in the Kosice region aged 18–55, and the control group was the majority population in the same region and of the same age composition. The majority population was divided into two subgroups: the majority population with (46%) and without (54%) a Roma population living in the vicinity.

**Figure 1:**
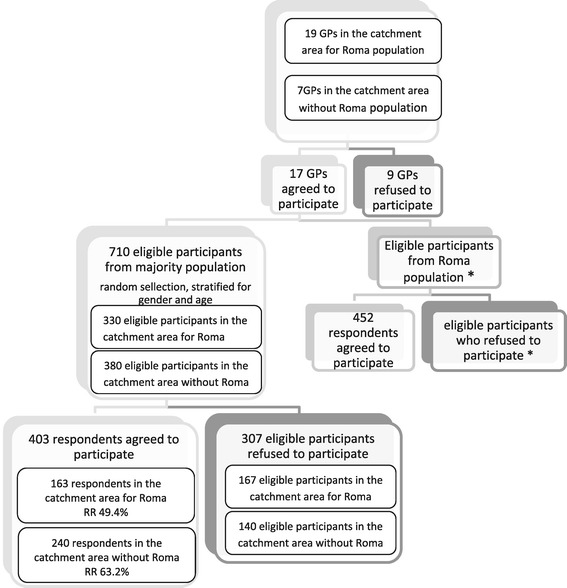
Recruitment of the Roma and non-Roma population.

We randomly selected separated or segregated Roma settlements with at least 500 inhabitants from each district of the Kosice Region. The separated type refers to a Roma population concentrated in a certain part of a town or village – either inside or on the outskirts; the segregated type refers to a settlement that is remote from towns and villages or separated from them by a physical barrier [7]. We then randomly selected and contacted 26 general practitioners from a list of general practitioners in the region. A Roma sample stratified in regard to gender and age was recruited directly in the settlements by cooperating with local Roma community workers. Of all Roma who were present in the settlements and received information about our study, 452 participated. Since the recruitment of Roma patients was carried out under the unpredictable conditions of Roma settlements, we were unable to record and compute the response rate. Respondents from the majority population were randomly chosen from a list of patients provided by the general practitioners. These patients were contacted via phone and mail by trained research assistants who provided information about our study and invited them to participate. Further details of recruitment of the Roma and non-Roma population can be seen in Figure 1.

Detailed information about our study and its procedures was given to all patients and informed consent was signed prior to the medical check-ups. Trained medical personnel collected the blood and urine samples and performed anthropometric measures in the surgeries of the cooperating general practitioners. For the majority population, trained assistants were present in the surgeries to assist with the questionnaires, if needed. Roma respondents were interviewed individually by trained community workers and assistants in community centres. We used an assisted self-administered interview adapted from other methods of collecting survey data, which seems to have the smallest impact on data reliability [27]. The study was approved by the Ethics Committee of the Medical Faculty at Safarik University in Kosice. Participation in the study was fully voluntary and anonymous. Inclusion criteria for the respondents were as follows: no preventive medical check-up in the past two years, no acute illness, appropriate age and be able to take a time off from the work during the week of data collection to be present in the surgery of their general practitioner. The final sample comprised 452 Roma (mean age = 34.7; SD = 9.14; 35.2% men) and 403 (mean age = 33.5; SD = 7.4; 45.9% men) non-Roma respondents.

### Measures

*Perceived health care accessibility problems* were measured by asking respondents the question: “How hard is it for you to find and ensure the necessary health services?” Possible responses were: *It is not difficult. / It is difficult but manageable. / It is not manageable without help of other person*. We merged the last two categories into one category: *It is difficult*.

*Perceived Social Support* (PSS) was measured using the Perceived Social Support Scale (PSSS) [28], which is a 12-item self-reported questionnaire assessing perceived social support in three dimensions (from family, friends and significant others). For the purpose of our study we used the subscale for family (4 items) and friends (4 items). A 7-point Likert-type format was used ranging from totally disagree (1) to totally agree (7). The range of sum scores in each dimension was 4–28. The higher respondents scored in a particular dimension, the higher were their levels of perceived social support. Both subscales showed satisfactory internal consistency (Cronbach’s α = 0.89 for the family; Cronbach’s α = 0.90 for friends).

*Health care specific Social Support* (HCSSS) was measured by asking respondents the question: Who can help you when you need to find and ensure the necessary health services (tick all boxes that apply to you)? Possible responses were: Family/Friends, acquaintances and neighbours/Community workers (health, field, social)/Mayor/Priest/Nobody, I always have to help myself/Other, please specify… For the purpose of our study we used only the first two categories to explore whether they correspond with the family and friends subscales from the Perceived Social Support Scale [28].

*Highest education* as an indicator of socioeconomic position was measured by asking respondents the question: “What is your highest educational degree attained?” Possible responses were: *Unfinished elementary/Finished elementary/Apprenticeship/Secondary/University*. We merged the first two categories into one category: *Elementary* and the last two categories into one category: *Higher* education.

### Statistical analyses

First we used chi-square statistics and the Mann–Whitney U test to explore the differences between Roma and non-Roma regarding independent and outcome variables (Table 1). Next, we used logistic regression analysis to assess differences in perceived health care accessibility problems by ethnicity and the degree to which social support could account for these ethnic differences (Tables 2 and 3). We tested the crude effects of perceived health care accessibility problems and each of the included covariates (gender, age, and general social support from family and friends), respectively (Table 2). Next, we tested the association of perceived health care accessibility problems with ethnicity, subsequently adjusted for gender, age (Table 3, Model 1) and social support (PSS) of family and friends (Model 2). We also tested the association of perceived health care accessibility problems with ethnicity, subsequently adjusted for gender, age and health care specific social support (HCSSS) of family and friends (Model 3). Model 4 tested the association of perceived health care accessibility problems with ethnicity adjusted for gender, age and all four types of social support together. In additional analyses we also adjusted all models for highest education (not shown). We also assessed the interactions between ethnicity and the PSS of family (Table 4, Model 1), friends (Model 2), HCSSS of family (Model 3) and friends (Model 4) adjusted for the main effects, gender and age. All data were analysed using IBM SPSS 20 for Windows.

**Table 1:** **Distribution of covariates among Roma and non-Roma**

	**non-Roma**		**Roma**		**Total**		Roma vs. non-roma p-value*
	**N = 403**	**(%)**	**N = 452**	**(%)**	**N = 855**	**(%)**	
**Gender**							<.001^a^
Men	185	45.9	159	35.2	344	40.2	
Women	218	54.1	293	64.8	511	59.8	
**Perceived health care accessibility (Total)**							<.001^a^
Not difficult	299	78.5	225	50.9	524	63.7	
Difficult but manageable	73	19.2	143	32.4	216	26.2	
Not manageable without help	9	2.4	74	16.7	83	10.1	
***Perceived health care accessibility in men***							<.001^a^
Not difficult	140	79.1	86	55.5	226	68.1	
Difficult but manageable	31	17.5	40	25.8	71	21.4	
Not manageable without help	6	3.4	29	18.7	35	10.5	
***Perceived health care accessibility in women***							<.001^a^
Not difficult	159	77.9	139	48.4	298	60.7	
Difficult but manageable	42	20.6	103	35.9	145	29.5	
Not manageable without help	3	1.5	45	15.7	48	9.8	
**Highest education**							<.001^a^
Higher	300	76.3	10	2.3	310	37.1	
Apprenticeship	84	21.4	73	16.5	157	18.8	
Elementary	9	2.3	360	81,3	369	44.1	
**Health care specific social support (HCSSS)**							
Family	328	81.4	368	81.4	696	81.4	ns
Friends	144	35.7	121	26.8	265	31.0	<.01^a^
	Mean (SD)		Mean (SD)				
**Social support (PSSS)**							
Family	24.00 (5.12)		24.15 (5.71)		N = 792		<.05^b^
Friends	22.90 (5.01)		20.77 (7.31)		N = 785		<.01^b^

*statistically significant differences between Roma and non-Roma.

^a^Chi-square tests.

^b^Mann–Whitney U test.

ns – not significant.

**Table 2:** **Crude effects of perceived health care accessibility problems and each of the included covariates (gender, age, general social support (PSSS) from family and friends and health care specific social support (HCSSS) from family and friends), respectively: odds ratios (OR) and 95% confidence intervals (CI)**

**Health care accessibility problems**		**Model 1**
		**OR (95% CI)**
		**Crude effects**
Ethnicity	*Non-Roma*	1 (reference)
	*Roma*	3.52 (2.59-4.78)***
Gender	*Women*	1 (reference)
	*Men*	0.72 (0.54-0.97)*
Age		1.02 (1.00-1.04)*
Social support (PSSS)	*Family*	0.96 (0.93-0.98)***
	*Friends*	0.96 (0.93-0.98)***
Social support (HCSSS)	*Family*	0.85 (0.59-1.24)
	*Friends*	0.65 (0.47-0.89)**
Highest education	*Higher*	1 (reference)
	*Apprenticeship*	2.42 (1.58-3.71)***
	*Elementary*	3.51 (2.49-4.96)***

*p < .05, **p < .01, ***p < .001.

**Table 3:** **Associations of perceived health care accessibility problems with ethnicity subsequently adjusted for gender, age (Model 1), social support (PSSS) of family (Model 2), friends (Model 3) and both together (Model 4): odds ratios (OR) and 95% confidence intervals (CI)**

**Health care accessibility problems**		**Model 1**	**Model 2**	**Model 3**	**Model 4**
		**OR (95% CI)**	**OR (95% CI)**	**OR (95% CI)**	**OR (95% CI)**
Ethnicity	*Non-Roma*	1 (reference)	1 (reference)	1 (reference)	1 (reference)
	*Roma*	3.21 (2.33-4.43)***	3.16 (2.27-4.39)***	3.35 (2.45-4.58)***	3.12 (2.24-4.34)***
Gender	*Women*	1 (reference)	1 (reference)	1 (reference)	1 (reference)
	*Men*	0.80 (0.58-1.11)	0.80 (0.57-1.10)	0.86 (0.63-1.17)	0.81 (0.58-1.12)
Age		1.01 (0.99-1.03)	1.01 (1.00-1.03)	1.01 (1.00-1.03)	1.01 (1.00-1.03)
Social support (PSSS)	*Family*		0.96 (0.93-0.99)**		0.96 (0.93-0.99)**
	*Friends*		0.98 (0.95-1.00)		0.98 (0.96-1.01)
Health care specific social support (HCSSS)	*Family*			0.95 (0.64-1.41)	1.03 (0.67-1.58)
	*Friends*			0.74 (0.53-1.04)	0.77 (0.54-1.10)

**p < .01, ***p < .001.

**Table 4:** **The effect of interaction between Ethnicity and Social support (PSSS) of family (Model 1), friends (Model 2), health care specific social support (HCSSS) of family (Model 3) and friends (Model 4) on Perceived health care accessibility problems adjusted for main effects, Gender and Age: odds ratios (OR) and 95% confidence intervals (CI)**

**Health care accessibility problems**		**Model 1**	**Model 2**	**Model 3**	**Model 4**
		**OR (95% CI)**	**OR (95% CI)**	**OR (95% CI)**	**OR (95% CI)**
Ethnicity	*Non-Roma*	1a	1a	1a	1a
	*Roma*	2.43 (0.61-9.64)	0.44 (0.13-1.46)	1.66 (0.82-3.38)	2.91 (2.02-4.21)***
Social support (PSSS)	*Family*	0.94 (0.90-0.99)**			
	*Friends*		0.90 (0.86-0.95)***		
Social support (HCSSS)	*Family*			0.52 (0.28-0.96)*	
	*Friends*				0.55 (0.32-0.95)*
(PSSS) *Family social support by Ethnicity*		1.01 (0.96-1.07)			
(PSSS) *Friends social support by Ethnicity*			1.09 (1.04-1.16)***		
(HCSSS) *Family social support by Ethnicity*				2.41 (1.10-5.30)*	
(HCSSS) *Friends social support by Ethnicity*					1.62 (0.81-3.24)

*p < .05, **p < .01, ***p < .001.

## Results

There are significantly more women, those who reported health care accessibility problems and those with lower education among Roma in comparison with non-Roma (Table 1). Roma perceive significantly more PSS from their family and significantly less PSS from their friends in comparison with non-Roma (Table 1). We did not find any ethnic differences in regard to health care specific social support from the family (Table 1). Conversely, non-Roma perceive significantly more health care specific social support from their friends when compared with Roma (Table 1).

Roma have a 3.5-times higher chance of reporting health care accessibility problems in comparison with non-Roma (Table 2). There is also a slightly higher chance of reporting health care accessibility problems among women compared with men (Table 2). The likelihood of reporting health care accessibility problems increases with increasing age (Table 2). Social support (PSS) in both dimensions significantly decreased the likelihood of reporting health care accessibility problems (Table 2). Health care specific social support (HCSSS) from family significantly decreases the likelihood of reporting health care accessibility problems (Table 2), whereas the effect of HCSSS from friends is not significant.

Adding PSS from family and friends into the model decreased the association between health care accessibility problems and Roma ethnicity (Table 3, Model 2). Adding HCSSS from family into the model increased the association between health care accessibility problems and Roma ethnicity (Table 4, Model 3). The results from the final model indicate that family is a dimension of social support which partially decreases the likelihood of reporting health care accessibility problems (Table 3, Model 4). Adjustment for highest education hardly affected the observed associations (not shown), because there is a strong mutual association between ethnicity and highest level of education which showed effects of collinearity; thus, these two constructs measure practically the same variable.

Since we found statistically significant ethnic differences regarding perceived health care accessibility problems and social support, we also assessed whether the effect of PSS and HCSSS on the likelihood of reporting health care accessibility problems was modified by ethnicity (Table 4), and this showed a statistically significant interaction effect in regard to PSS from friends (Table 4, Model 2) and HCSSS from family (Table 4, Model 3). The significant interaction of ethnicity with PSS from friends indicates an increasing effect of ethnicity on health care accessibility problems with decreasing levels of PSS (Table 4, Model 2). Thus, non-Roma who perceive lower levels of social support from their friends have a higher chance of reporting health care accessibility problems in comparison with Roma. The significant interaction of ethnicity with HCSSS from family indicates an increasing effect of ethnicity on health care accessibility problems with decreasing levels of HCSSS (Table 4, Model 2). Thus, non-Roma who perceive lower levels of HCSSS from their family have a higher chance of reporting health care accessibility problems in comparison with Roma.

## Discussion

The aim of this study was to explore the mediating as well as moderating effect of social support on the association between health care accessibility problems and ethnicity. Roma, in comparison with non-Roma, have a more than 3-times higher chance of reporting health care accessibility problems. Perceived social support (PSS) from family and friends decreases the likelihood of reporting health care accessibility problems in both Roma and non-Roma, whereas the family seems to be a more important factor. Health care specific social support (HCSSS) from family also decreases the likelihood of reporting health care accessibility problems, but only in non-Roma. There is no significant difference between Roma and non-Roma in how PSS from family affects health care accessibility problems. However, the lack of PSS from friends as well as HCSSS from family seems to increase the likelihood of health care accessibility problems more in non-Roma than in Roma.

We found no differences between Roma and non-Roma regarding HCSSS from family. However, the effect of HCSSS from family on the likelihood of reporting health care accessibility problems was modified by ethnicity, as the interaction was significant. These results suggest that the effect of HCSSS from family on health care accessibility problems differs between Roma and non-Roma. On the other hand, Roma and non-Roma differed in regard to PSS from family. The effect of PSS from family on the likelihood of reporting health care accessibility problems was not modified by ethnicity, as the interaction was not significant. Thus, it is likely that Roma and non-Roma do not differ in how general social support affects their health care accessibility problems. In line with this, several other studies have also confirmed the role of family support as an important factor in regard to influencing health care-seeking behaviour or treatment adherence [22-24].

We found statistically significant differences between Roma and non-Roma regarding HCSSS from friends. The effect of HCSSS from friends on the likelihood of reporting health care accessibility problems was not modified by ethnicity. Roma and non-Roma also differed in regard to PSS from friends. Moreover, the effect of PSS from friends on the likelihood of reporting health care accessibility problems was modified by ethnicity, as the interaction was significant. Thus it is likely that Roma and non-Roma differ in how general social support affects their health care accessibility problems.

We found that social support from family and friends has a positive/protective effect on health care accessibility problems, which means that people with higher levels of family or friend support are less likely to report problems. Of all the types of social support we explored, PSS from family is the most important factor for helping to overcome health care accessibility problems, regardless of ethnicity. However, it does not substantially decrease the ethnic differences in health care accessibility problems. Roma in Slovakia are not likely to participate in a wider network of relationships [29]. They have limited access to structures beyond family ties and their local community, which are intertwined by close and distant family relationships [29]. In our opinion, one of the possible explanations is that for the majority population the wider social network (e.g. friends, acquaintances, and community) serves as an additional support in order to find and arrange the best possible and most accessible care of a particular doctor in a particular field of medical care. This is in line with the informational function of social support (e.g. providing advice, suggestion, directives, information) as identified by House and Kahn (1985). On the other hand, we assume that Roma usually do not have such friends or acquaintances who can advise them about health care providers or solutions for their health related problems within the health care system.

The health of Roma is perceived through the perspective of their families, which seems to be rather logical considering the tight social bonds within Roma families and communities [15]. Different social norms, values, health beliefs and behavioural differences [13,14,30] may also lead to different perception of health as well as different ways of using or not using health care services [12,31]. Roma often prefer home treatment with their own curative methods and/or have had bad experiences with the health care system [12,32]. Moreover, Roma themselves often cite their own bad economic situation (lack of money for medication and transportation) as justification for the problematic use of health care services [12]. Nevertheless, the worse health of Roma can be partially accounted for by worse access to health care services [12,32].

### Strengths and limitations

The strength of our study is that it comprises a considerable representative sample of a hard-to-reach population living in separated or segregated Roma settlements. We were able to compare them with the majority population living in the same geographical area, although our results should be generalised with caution, as Roma are a very heterogeneous group in terms of living conditions and levels of integration; thus, our results can be generalised only to the Roma population living in Roma settlements.

A limitation of our study may be that data from the Roma were collected via an interview, and data from the non-Roma came via self-reported questionnaires. The reason for this was to cope with illiteracy among the Roma, which we considered to be a more serious source of non-response and bias than using two different types of administration. In Roma, we used the technique of assisted self-administration, which has shown good reliability of data [27]. Moreover, using this technique turned out to be a good decision, as most of Roma were not able to fill in the questionnaires without assistance.

### Implication

As suggested by our results, the worse accessibility of health care in the Roma population creates a severe public health concern. This issue can be partially solved through promotion of social support, which appears to partially decrease health care accessibility problems equally among the majority population as well as in the Roma population. As Roma do not seem to effectively use potential support from wider social networks beyond family ties, we need to look for other resources which might ease health care accessibility for Roma. Health-related behaviours of individuals within Roma communities seem to be influenced by privileged males and elders, or family members even when their advice or beliefs might be inappropriate [33]. One of the possible options for benefiting from this is to use Roma health mediators and community workers to identify influential individuals who are able to change a community’s fear and distrust and persuade and teach Roma to seek out and appropriately use health care services [34,35]. Moreover, making access to health care easier for one Roma individual creates benefits for the whole community via social support. Social support might potentially promote population health beyond the targeted individuals by influencing the health of others through existing social networks [36]. Future research should focus on a broader range of factors affecting Roma access to the health care system and proper validation of measures aimed at accessibility of health care being introduced into the practice.

## Conclusions

Perceived social support from family is the most important factor which helps to overcome health care accessibility problems regardless of ethnicity. The lack of perceived social support from friends as well as health care specific social support from family seems to increase the likelihood of health care accessibility problems more in non-Roma than in Roma. As Roma do not seem to effectively use potential support from wider social networks beyond family ties, we need to look for other resources which might ease health care accessibility for Roma.
